# The Contribution of Orai(CRACM)1 and Orai(CRACM)2 Channels in Store-Operated Ca^2+^ Entry and Mediator Release in Human Lung Mast Cells

**DOI:** 10.1371/journal.pone.0074895

**Published:** 2013-09-10

**Authors:** Ian Ashmole, S. Mark Duffy, Mark L. Leyland, Peter Bradding

**Affiliations:** 1 Department of Infection, Immunity and Inflammation, Institute for Lung Health, University of Leicester, Glenfield Hospital, Leicester, Leicestershire, United Kingdom; 2 Department of Biochemistry, Henry Wellcome Building, University of Leicester, Leicester, Leicestershire, United Kingdom; Cornell University, United States of America

## Abstract

**Background:**

The influx of extracellular Ca^2+^ into mast cells is critical for the FcεR1-dependent release of preformed granule-derived mediators and newly synthesised autacoids and cytokines. The Orai(CRACM) ion channel family provide the major pathway through which this Ca^2+^ influx occurs. However the individual role of each of the three members of the Orai channel family in Ca^2+^ influx and mediator release has not been defined in human mast cells.

**Objective:**

To assess whether there might be value in targeting individual Orai family members for the inhibition of FcεRI-dependent human lung mast cells (HLMC) mediator release.

**Methods:**

We used an adenoviral delivery system to transduce HLMCs with shRNAs targeted against Orai1 and Orai2 or with cDNAs directing the expression of dominant-negative mutations of the three known Orai channels.

**Results:**

shRNA-mediated knockdown of Orai1 resulted in a significant reduction of approximately 50% in Ca^2+^ influx and in the release of β-hexosaminidase (a marker of degranulation) and newly synthesized LTC_4_ in activated HLMCs. In contrast shRNA knockdown of Orai2 resulted in only marginal reductions of Ca^2+^ influx, degranulation and LTC_4_ release. Transduced dominant-negative mutants of Orai1, -2 and -3 markedly reduced Orai currents and completely inhibited HLMC degranulation suggesting that Orai channels form heteromultimers in HLMCs, and that Orai channels comprise the dominant Ca^2+^ influx pathway following FceRI-dependent HLMC activation. Inhibition of Orai currents did not alter HLMC survival. In addition we observed a significant down-regulation of the level of CRACM3 mRNA transcripts together with a small increase in the level of CRACM1 and CRACM2 transcripts following a period of sustained HLMC activation.

**Conclusion and Clinical Relevance:**

Orai1 plays an important role in Ca^2+^ influx and mediator release from HLMCs. Strategies which target Orai1 will effectively inhibit FcεRI-dependent HLMC activation, but spare off-target inhibition of Orai2 in other cells and body systems.

## Introduction

Mast cells play a critical role in the development of asthma and related allergic diseases [1]. Mast cell activation leads to the release of a battery of mediators including preformed granule-derived mediators such as histamine and proteases and newly synthesized prostaglandins, leukotrienes and cytokines. Excess release of these mediators as a result of aberrant activation contributes to allergic disease states. FcεRI-dependent activation of mast cells is characterised by an influx of extracellular Ca^2+^ that is essential for mediator release. A major pathway through which this influx occurs is through Ca^2+^ release activated Ca^2+^ (CRAC) channels, also known as store-operated channels. These channels are activated by the inositol 1,4,5-triphosphate (IP_3_)-mediated depletion of the endoplasmic reticulum (ER) Ca^2+^ stores that occurs following cell surface receptor-dependent activation of phospholipase C [2].

CRAC channels were first characterised in rodent mast cells twenty years ago [3,4], but the molecular components of the CRAC channel were only recently identified. STIM1 acts as the sensor of the ER Ca^2+^ concentration and transmits this information to the CRAC channel pore [5]. Orai1 (also known as CRACM1) was subsequently identified as the Ca^2+^-selective pore forming protein in the plasma membrane [6–10]. Two further homologues are expressed in mammalian cells, Orai2 and Orai3. These show a high degree of sequence homology with Orai1 but have distinct functional properties [11,12]. Heterodimerisation between Orai channel subunits has been reported in heterologous expression systems [11,13]. It is not yet known whether this also occurs in mast cells. Orai channels are essential for both human and rodent mast cell mediator release. Ca^2+^ influx, degranulation, leukotriene (LT)C_4_ release and TNFα production are all greatly reduced in foetal liver-derived mast cells from a Orai1 knockout mouse [14]. Similarly we demonstrated that block of Orai channels in human lung mast cells (HLMCs) using the specific blockers Synta-66 and GSK-7975A reduced Ca^2+^ influx, degranulation, LTC_4_ release and cytokine secretion [15].

Human and rodent mast cells express all three Orai subunits at the mRNA level [14,15]. However the relative contribution of these channels to Ca^2+^ influx in human mast cells is not currently known as current Orai blockers inhibit all family members. In mast cells derived from the mouse Orai1 knockout, Ca^2+^ influx was reduced by 70% with the remaining Ca^2+^ influx blocked by Orai channel inhibitors suggesting that Orai2 and/or Orai3 also contribute to Ca^2+^ influx in rodent mast cells [14]. However, the substantial differences evident between rodent and human mast cells mean that it cannot be assumed that this is the case in human mast cells. Understanding the role of individual Orai family members in HLMCs is important because the development of pharmacological strategies that target individual family members will reduce off-target inhibition of other Orai family members in other cells and body systems. In this study we therefore investigated the relative contributions of Orai channels to Ca^2+^ influx and mediator release in HLMCs.

## Methods

### Ethics statement

All patients donating tissue gave written informed consent and the study was approved by the National Research Ethics Service (reference 07/MRE08/42).

### Human mast cell purification and cell culture

HLMCs were purified from macroscopically normal human lung obtained within one hour of resection for lung cancer [16] and cultured as previously described [17]. Final mast cell purity was >99% and viability >97%. Cells were activated with an anti-FcεR1α antibody (Fisher Scientific) as required.

### Adenoviral Transduction of HLMC

Adenoviruses (Ad5C20Att01) encoding shRNA directed against human Orai1, Orai2, Orai3 and luciferase, or directing the expression of GFP, were purchased from Biofocus Ltd (Leiden, The Netherlands). Optimal conditions for knockdown of Orai transcripts in HLMC were determined empirically for each virus 2-7 days post infection, at a multiplicity of infection of 10-250. Knockdown efficiency was determined by quantitative RT-PCR. Satisfactory knockdown of transcripts was observed 4 days post infection using a multiplicity of infection of 100 (Orai1 and -2) or 250 (Orai3). This time period was used for all subsequent experiments. Matched experiments using a virus expressing shRNA targeted against luciferase or expressing GFP were used to control for nonspecific effects of adenoviral infection on Orai channel expression and cell viability.

Adenoviruses (Ad5C20Att01) expressing dominant-negative pore mutations of Orai1 (E106Q), Orai2 (E80Q) and Orai3 (E81Q) were prepared by Biofocus Ltd using cDNAs supplied by our laboratory. Mutants were generated by the Quik Change method (Stratagene) using Orai1-3 cDNAs cloned into vector pcDNA3 as template. Mutants were verified by sequencing of the entire mutant cDNAs.

### Quantitative RT-PCR

Total RNA was isolated from cells using an RNAqueous-Micro kit (Applied Biosystems, Warrington, United Kingdom) according to the manufacturer’s instructions. Approximately 100 ng of total RNA was used to generate cDNA using random decamer primers and a Retroscript kit (Applied Biosystems). TaqMan probes for human Orai1 (Hs00385627_m1), Orai2 (Hs00259863_m1), Orai3 (Hs00752190_s1) and beta-actin (Hs99999903_m1) together with TaqMan Gene Expression Master Mix (all Applied Biosystems) were used for quantification of Orai transcripts. Reactions were run on a Stratagene Mx3000P (Agilent Technologies, Stockport, United Kingdom) real time thermocycler.

### Cell survival assay

HLMCs were plated at 5 x 10^4^ cells/well of a 24 well plate in 1 ml culture medium. Recombinant adenovirus was added as appropriate and cells incubated for 96 hours. Cells were pelleted and then resuspended in 20 µl DMEM. An equal volume of trypan blue was added and cells counted in a haemocytometer. Exclusion of trypan blue was used to assess cell viability.

### Patch clamp electrophysiology

The whole-cell variant of the patch-clamp technique was used [18,19]. Patch pipettes were made from borosilicate fiber-containing glass (Clark Electromedical Instruments, Reading, UK), and their tips were heat polished, typically resulting in resistances of 4–6 MΩ. The standard pipette solution contained (in mM): CsCl (140), MgCl_2_ (2), HEPES (10), NaATP (2) GTP (0.1) EGTA (5); pH 7.3 with KOH. Prior to use 30 µM IP_3_ was added to the pipette solution. The standard external solution contained (in mM), NaCl (140), KCl (5), CaCl_2_ (2), MgCl_2_ (1), HEPES (10) and glucose (5); pH 7.3 with NaOH. For recording, mast cells were placed in 35 mm dishes containing standard external solution.

Whole cell currents were recorded using an Axoclamp 200A amplifier (Axon Instruments, Foster City, CA, USA), and currents usually evoked by applying voltage commands to a range of potentials in 10 mV steps from a holding potential of -20 mV. The currents were digitised (sampled at a frequency of 10 kHz), stored on computer and subsequently analysed using pClamp software (Axon Instruments). Capacitance transients were minimised using the capacitance neutralisation circuits on the amplifier. Correction for series resistance was not routinely applied. Experiments were performed at 27°C, temperature being controlled by a Peltier device. Experiments were performed with a perfusion system (Automate Scientific Inc, San Francisco, CA, USA) to allow solution changes, although drugs were added directly to the recording chamber. Currents in some experiments were also evoked using a ramp protocol consisting of a continuous voltage ramp from -120 mV to +120 mV.

### Mediator assays

β-hexosaminidase release was used as a measure of degranulation. 2.5x10^4^ HLMCs in a volume of 90 µl were added to a 96 well V bottom plate in duplicate. Plates were pre-incubated at 37°C for 10 min before activation of cells by the addition of 10 µl 10x anti-FcεRIα antibody (final dilution of antibody 1:300). Cells were incubated at 37°C for 30 min and then either centrifuged, and the supernatant decanted for measurement of mediator content or lysed in 1% Triton X-100 (Sigma) for the determination of total β-hexosaminidase content. 40µl of supernatant or cell lysate were incubated in triplicate with 80µl 4-nitrophenyl N-acetyl-β-D-glucosaminide (Sigma) in 0.05M citrate buffer pH4.5 in a 96 well plate at 37°C for 1-2 hours. The reaction was terminated by the addition of 200µl carbonate buffer pH10.0 and absorbances determined at 405 nm in a microplate reader (Wallac). Net β-hexosaminidase release was expressed as a percentage of the total β-hexosaminidase content. LTC_4_ release was measured by ELISA (Cambridge Bioscience Ltd, Cambridge, United Kingdom).

### Statistical analysis

Data were compared using paired or unpaired t test and ANOVA or repeated measures ANOVA as appropriate, using GraphPad Prism 5 software. P<0.05 was considered to be statistically significant. N numbers represent number of HLMCs (from at least 3 independent donors) used for patch clamp experiments, or the number of independent experiments from different donors for mRNA expression, mediator release and viability experiments.

## Results

### Orai1 but not Orai2 plays a major role in Ca^2+^ influx in HLMC

**Figure 1:**
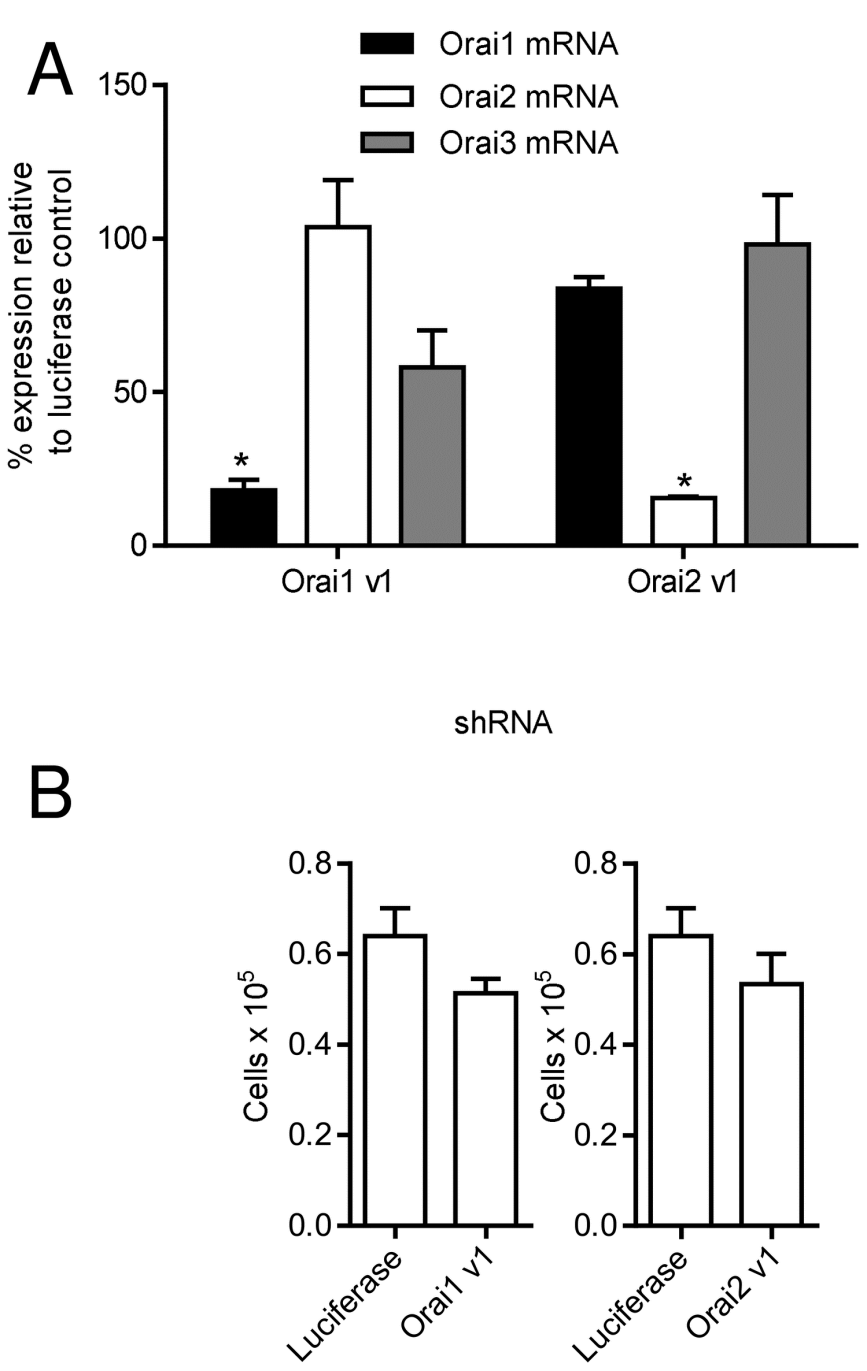
Knockdown of Orai channels using recombinant adenoviruses expressing shRNA. (**A**) Knockdown efficiency of shRNAs against Orai1 (Orai1 v1) and Orai2 (Orai2 v1) was determined in HLMCs 4 days following transduction with virus by quantitative RT-PCR (n=3 independent experiments using HLMCs from 3 donors), * p<0.05 compared to expression in cells transduced with luciferase control. All other conditions were not significantly different to control. (**B**) Transduction of HLMCs with adenoviruses expressing shRNAs against Orai1 (Orai1 v1) and Orai2 (Orai2 v1) (n=3 donors), had no significant effect on cell viability after 4 days compared to luciferase control virus.

We used an adenoviral delivery system to transduce HLMCs with shRNAs targeted against Orai1, -2 and -3, or against luciferase as a control. The shRNAs were effective in downregulation of the mRNAs for Orai1 and -2 in HLMCs 4 days following transduction with virus, as shown by quantitative RT-PCR (Figure **1A**). Knockdown of Orai3 transcripts however required the use of a multiplicity of infection (moi) of adenovirus (moi 250), significantly higher than that required to achieve knockdown of Orai1 and Orai2 transcripts (moi 100). However, at 250 MOI Orai3 and luciferase control transfected cells appeared unhealthy in that they could not be patch clamped and exhibited reduced mediator release (see below). Interpreting the functional effects of Orai3 knockdown was therefore considered unreliable.

The level of Orai3 transcripts was also reduced in cells transduced with shRNA targeted against Orai1, although this did not reach statistical significance and was markedly less than knockdown of Orai1 mRNA (Figure **1A**). The expression of shRNAs against Orai1 and -2 did not affect cell viability monitored by Trypan blue exclusion over the time course of these experiments, compared to cells expressing the luciferase shRNA control (Figure **1B**).

**Figure 2:**
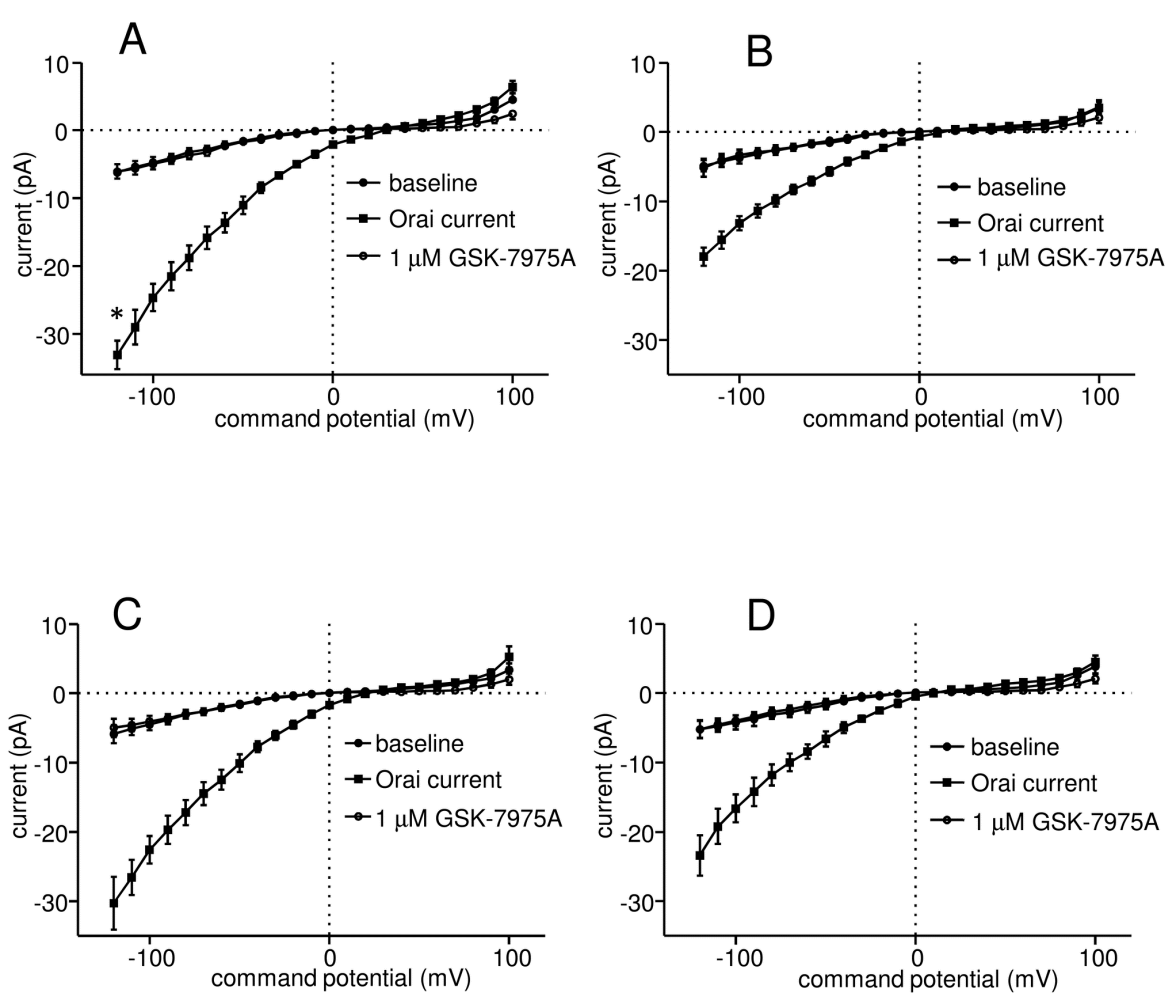
Knockdown of Orai1 but not Orai2 significantly reduces Ca^2+^ influx in activated HLMCs. Whole cell patch clamp current-voltage (I–V) curves from HLMCs transduced with recombinant adenoviruses expressing (A) shRNA against luciferase (n=19 cells), or (**B**) against Orai1 (n=13); and (**C**) shRNA against luciferase (n=11) or against Orai2 n=14 (**D**). (●) baseline current, (■) current elicited following dialysis of HLMCs with 30 µM IP_3_ for 4 min, (○) current following the addition of 1 µM GSK-7975A. Currents were recorded in 2 mM external Ca^2+^ and are shown as mean ± SEM. *p=0.0292 compared with corresponding curve in (**B**).

To elicit Orai currents in HLMCs IP_3_ was included in the patch pipette to deplete intracellular Ca^2+^ stores [2,15]. HLMCs transduced with the luciferase shRNA developed an inwardly rectifying current characteristic of that carried by Orai channels. These currents were both qualitatively and quantitatively similar to those we have previously observed from untransduced HLMCs [15]. In experiments to examine the effect of Orai1 knockdown, a mean baseline current of -6.2 ± 1.2 pA (n=19 cells) at -120 mV, with a reversal potential of -4.6 ± 2.7 mV, was recorded from control cells transduced with luciferase shRNA (Figure **2A**). After 4 min in the presence of IP_3_, these currents increased to -33.1 ± 2.1 pA, with a shift in reversal potential to 28.0 ± 4.4 mV (p<0.0001 compared to baseline for both current and reversal potential). We have previously observed in patch clamp experiments that addition of the Orai channel blocker GSK-7975A at a concentration of 1 µM to HLMCs resulted in the complete inhibition of Orai current in these cells [15]. Addition of 1 µM GSK-7975A similarly inhibited Ca^2+^ influx into IP_3_-activated HLMCs transduced with the control shRNA (Figure **2A**). Mean current at -120 mV was reduced to -6.1 ± 1.0 pA with a shift in reversal potential to -2.6 ± 1.9 mV (p<0.0001 for both current and reversal potential compared to control).

We observed a substantial reduction in the size of Ca^2+^ currents recorded from IP_3_-activated HLMCs transduced with shRNA directed against Orai1 compared to luciferase control (Figure **2B**). Baseline currents in these cells at -120 mV were -4.9 ± 0.8 pA, with a reversal potential of -9.3 ± 1.8 mV (n=13). Following 4 min in the presence of IP_3_, these currents increased to -18.0 ± 1.3 pA, with a shift in reversal potential to 16.7 ± 2.9 mV (p<0.0001 for current, p=0.00018 for reversal potential) (Figure **2B**). This represents a reduction in current to 48.6 ± 8.6% (p=0.0292) of the level of that recorded in control cells as a result of Orai1 knockdown (baseline currents subtracted). Currents generated by IP_3_ were again completely blocked following the addition of 1 µM GSK-7975A, being reduced to -5.2 ± 1.3 pA, with a reversal potential of -8.5 ± 2.1 mV (p<0.0001 for current, p = 0.00019 for reversal potential compared to post IP_3_ data) (Figure **2B**).

In contrast, we observed only a relatively small reduction in Orai currents in HLMCs transduced with the shRNA targeted against Orai2 compared to control which did not reach statistical significance (Figure **2C and D**). Mean baseline current at -120 mV recorded from HLMCs transduced with the control shRNA was -5.9 ± 1.3 pA, with a reversal potential of -7.0 ± 2.1 mV (n=11) (Figure **2C**), while that recorded from cells transduced with the Orai2 shRNA was -5.2 ± 1.2 pA, with a reversal potential of -8.9 ± 2.4 mV (n=14) (Figure **2D**). Following activation with IP_3_, currents at -120 mV increased to -30.3 ± 3.8 pA, with a shift in reversal potential to 21.4 ± 4.5 mV in control cells (p<0.0001 for both current and reversal potential) and to -23.4 ± 2.9 pA, with a reversal potential of 10.7 ± 3.2 mV in cells transduced with the Orai2 shRNA (p<0.0001 for current, p=0.00059 for reversal potential) (Figure **2C and D**). This represents a reduction in current to 74.5 ± 12.1% (p=0.3475) of the level of that recorded in control cells as a result of Orai2 knockdown (baseline currents subtracted). In both cases, currents generated by IP_3_ were completely blocked by the addition of 1 µM GSK-7975A (Figure **2C and D**).

HLMC transduced with shRNA targeted against Orai3 or luciferase control using virus at the higher moi of 250 were not possible to record in our patch clamp experiments due to the inability to obtain tight seals. Consequently we were unable to satisfactorily record Orai currents from these cells.

### Knockdown of Orai1 but not Orai2 attenuates HLMC mediator release

We next determined the effect of Orai channel knockdown on mast cell mediator release following FcεRIα-dependent activation by measuring the release of β-hexosaminidase as a marker of degranulation and LTC_4_ secretion as a marker of arachidonic acid metabolism. In experiments to assess the effect of Orai1 or Orai2 knockdown, net FcεRIα-dependent release of β-hexosaminidase from cells transduced with the luciferase shRNA control virus was 23.4 ± 4.2%, not significantly different to that observed from untreated cells (25.1 ± 4.2%, p=0.371, n=10 independent experiments with HLMC from different donors). FcεRIα-dependent release of LTC_4_ from control virus transduced cells was 741.6 ± 226.8 ng/10^6^ cells, again not significantly different from untreated cells (884.7 ± 446.9 ng/10^6^ cells, p=0.9599, n=5). In contrast, the higher moi used in experiments to assess the effects of Orai3 knockdown appeared to result in some non-specific effects, with both β-hexosaminidase and LTC_4_ secretion reduced in cells transduced with the luciferase control virus (β-hexosaminidase release 14.7 ± 0.7% with luciferase control, versus 31.4 ± 4.0%, untreated, p=0.0084, n=6; LTC_4_ secretion with luciferase control 651.6 ± 242.8 ng/10^6^ cells versus 1199.0 ± 450.2 ng/10^6^ cells untreated, p=0.0146, n=5). This again suggests that Orai3-transfected cells were not healthy, and it was therefore not possible to assess the contribution of Orai3 to mediator release.

**Figure 3:**
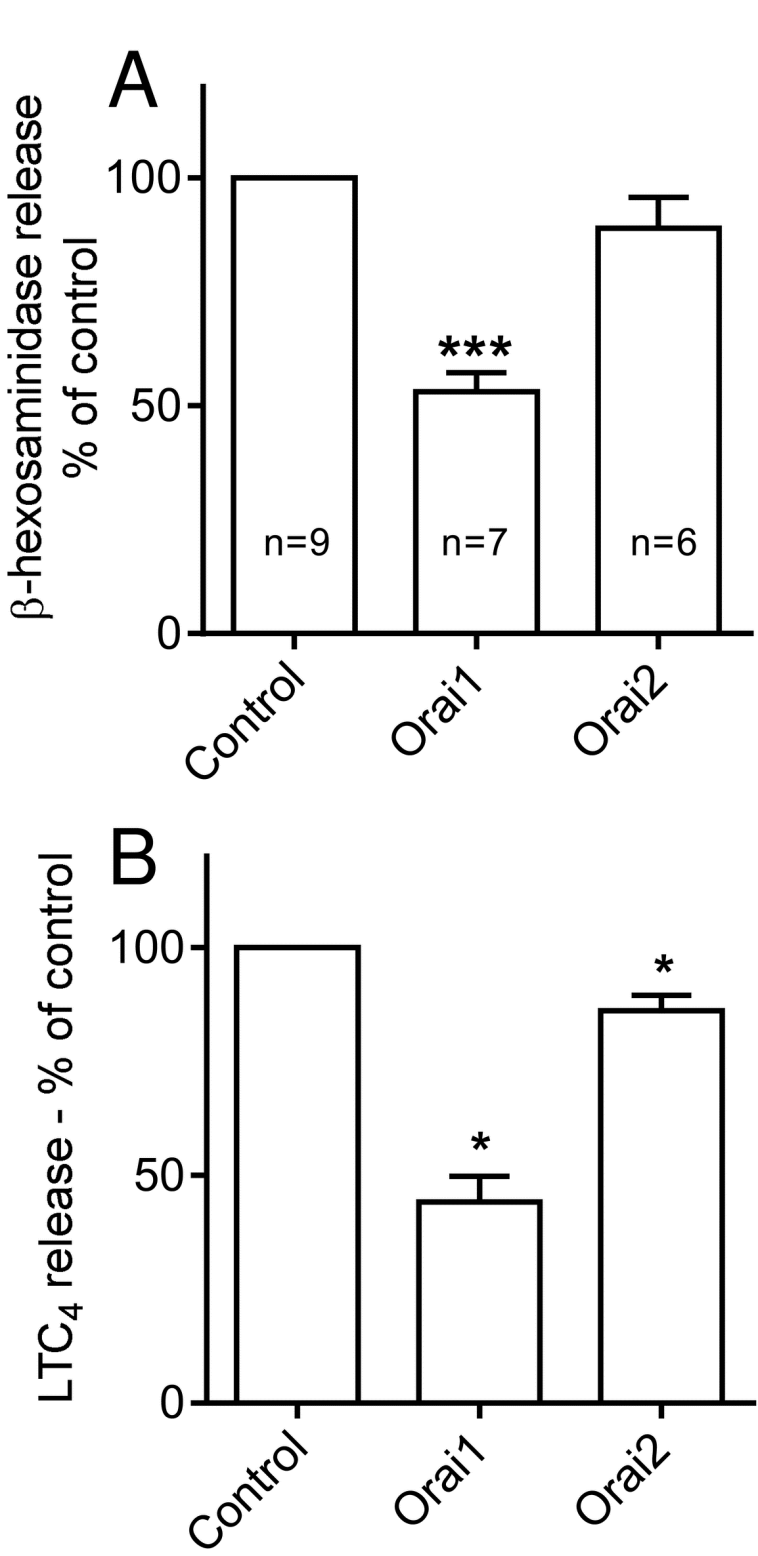
The effect of Orai channel knockdown on the release of HLMC (A) β-hexosaminidase (degranulation) and (B) LTC_4_. The graphs demonstrate the mean ± SEM release presented as a percentage of that observed in control cells transduced with adenovirus expressing shRNA directed against luciferase. n=6-10 independent donors for β-hexosaminidase, n=5 for LTC_4_. * p<0.05, *** p<0.001.

Knockdown of Orai1 markedly reduced FcεRIα-dependent β-hexosaminidase release to 53.1 ± 4.2% (n=9, p<0.0001) of that from control cells (Figure **3A**). In contrast only a slight reduction of β-hexosaminidase release was observed on knockdown of Orai2 (89.0 ± 6.7% of control cells, n=7, p=0.116) (Figure **3A**). A similar pattern of results was obtained on examining the effect of Orai channel knockdown on the release of LTC_4_ from activated HLMC. Knockdown of Orai1 resulted in a significant reduction of LTC_4_ release to 44.1 ± 5.7% (n=5, p=0.016) respectively of control cells (Figure **3B**). A much smaller but significant reduction was observed on knockdown of Orai2 (86.1 ± 3.5% of control cells, n=5, p=0.037) (Figure **3B**).

### Dominant-negative pore mutants of Orai channels abolish mast cell degranulation and greatly reduce Ca^2+^ influx

**Figure 4:**
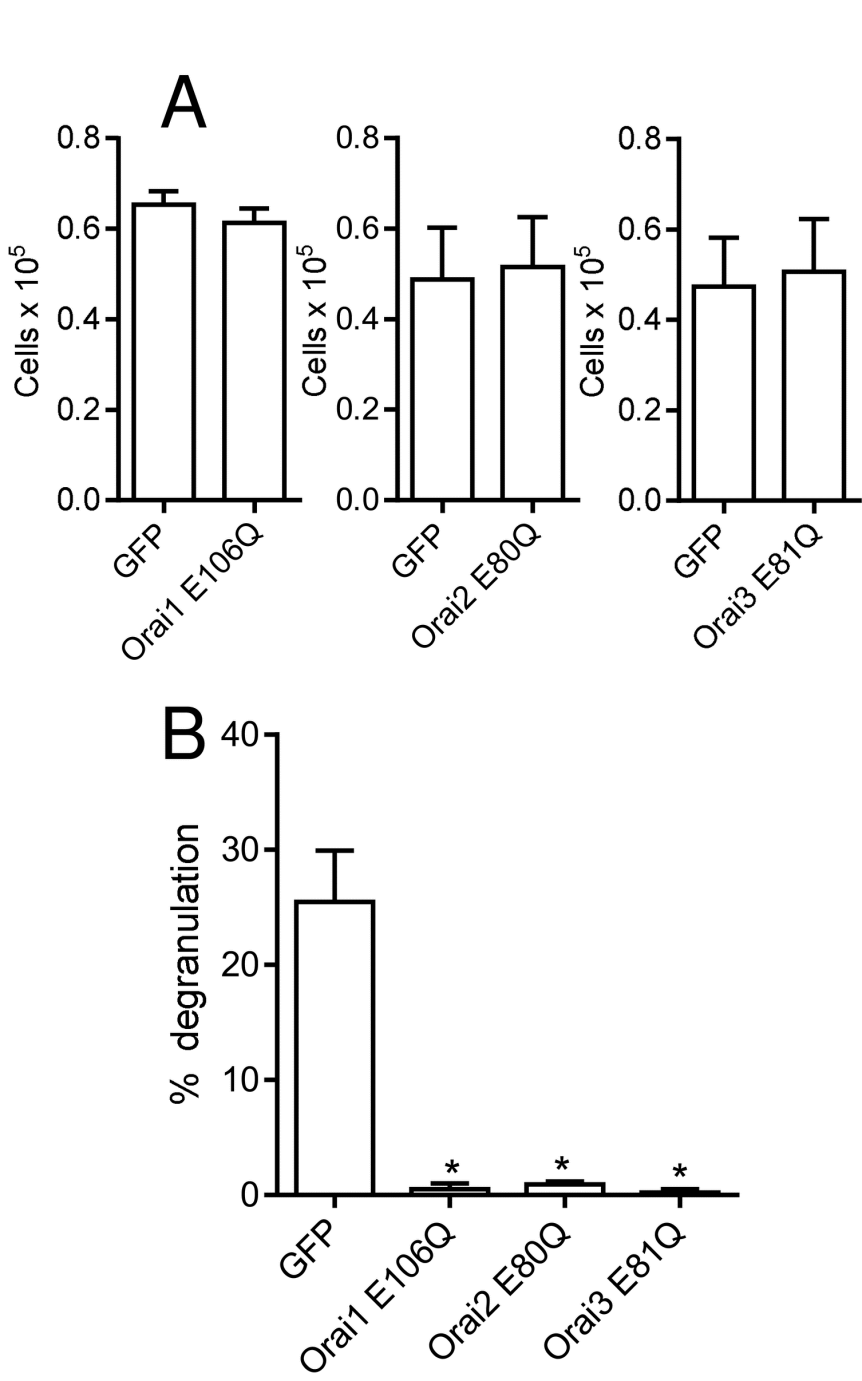
The effect of dominant negative mutations of Orai1 (E106Q), Orai2 (E80Q) and Orai3 (E81Q) on HLMC degranulation. (**A**) Transduction of HLMCs with Orai dominant-negative mutants had no effect on cell viability after 4 days compared to GFP control virus (n=5). (**B**) β-hexosaminidase degranulation was essentially abolished following transduction of HLMC with Orai dominant negative mutants. Mean ± SEM of net percentage release observed in cells transduced with adenoviruses expressing the indicated protein. n=4-7, * p<0.05 compared to GFP control.

To further characterise the composition of Orai channels in HLMCs, recombinant adenoviruses expressing a dominant negative pore mutant of Orai1 (E106Q) or the equivalent mutation in Orai2 (E80Q) and Orai3 (E81Q) were assembled and their effect on activated HLMCs assessed [20]. A recombinant virus directing the expression of GFP was used as a control. After 4 days of culture, there was no significant difference in HLMC viability between cells transduced with the GFP control and those transduced with the Orai1 E106Q expressing virus (n=5, p=0.312), the Orai2 E80Q virus (n=5, p=0.503), or the Orai3 E81Q virus (n=5, p=0.116) (Figure **4A**). This indicates that expression of the dominant negative Orai mutants had no effect on cell viability compared to the GFP control virus during this time period. β-hexosaminidase release from cells transduced with the GFP control virus was 25.5 ± 4.5%, (n=7), not significantly different from untreated cells (27.4 ± 4.8%, n=7, p=0.595). However β-hexosaminidase release from cells transduced with viruses expressing Orai1 E106Q (0.50 ± 0.47%, n=4), Orai2 E80Q (0.92 ± 0.22%, n=4), or Orai3 E81Q (0.22 ± 0.30%, n=4) was essentially abolished (Figure **4B**).

**Figure 5:**
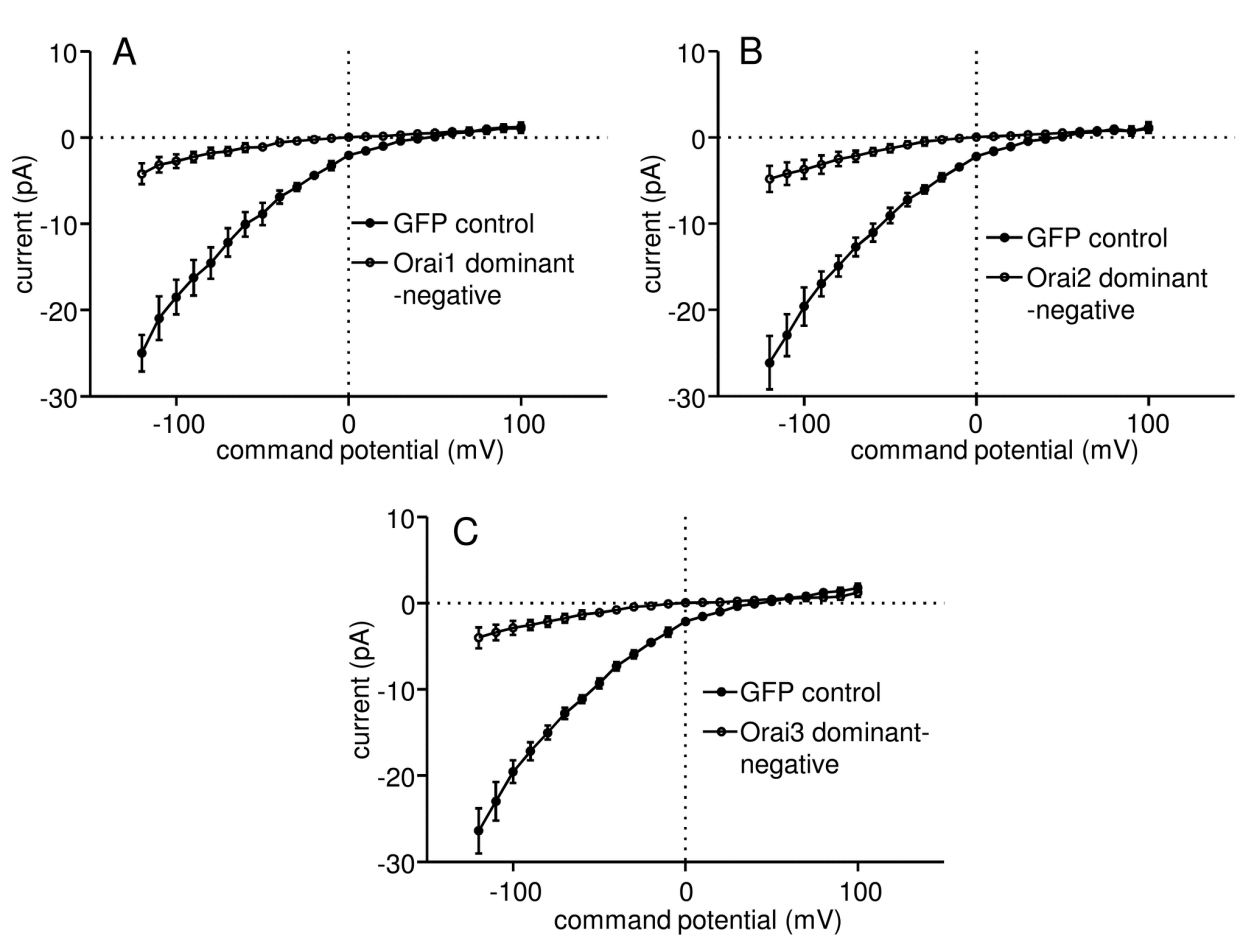
Expression of dominant negative mutations of Orai1-3 greatly reduce Ca^2+^ influx into activated HLMCs. Subtracted whole cell patch clamp current-voltage (I–V) curves from HLMCs transduced with recombinant adenoviruses expressing (**A**) GFP n=14 cells (●) or Orai1 E106Q n=15 (○), (**B**) GFP n=11 (●) or Orai2 E80Q n=14 (○), and (C) GFP n=11 (●) or Orai3 E81Q n=13 (○). Currents (mean ± SEM) were recorded in 2 mM external Ca^2+^ following dialysis with 3 µM IP_3_ for 4 min.

The expression of Orai dominant-negative channels also had a dramatic effect on Ca^2+^ influx into HLMCs measured using patch clamp electrophysiology (Figure **5**). Currents at -120 mV elicited by IP_3_ in HLMC transduced with the Orai1 E106Q mutant were only 16.8 ± 6.2% (p=0.00016) of those recorded in GFP control cells (baseline currents subtracted) (Figure **5A**). Similarly, currents at -120 mV were reduced to 18.3 ± 7.8% (p=0.0049) of the level in control cells as a result of expression of the Orai2 E80Q mutant (Figure **5B**); while currents at -120 mV in Orai3 E81Q expressing cells were 15.2 ± 6.1% (p=0.0049) of the level of control (Figure **5C**). The addition of 1 µM GSK-7975A to IP_3_-activated HLMC expressing either GFP, Orai1 E106Q, Orai2 E80Q or Orai3 E81Q resulted in a reduction of current back to baseline levels (not shown).

### Levels of Orai transcripts are altered by mast cell activation

**Figure 6:**
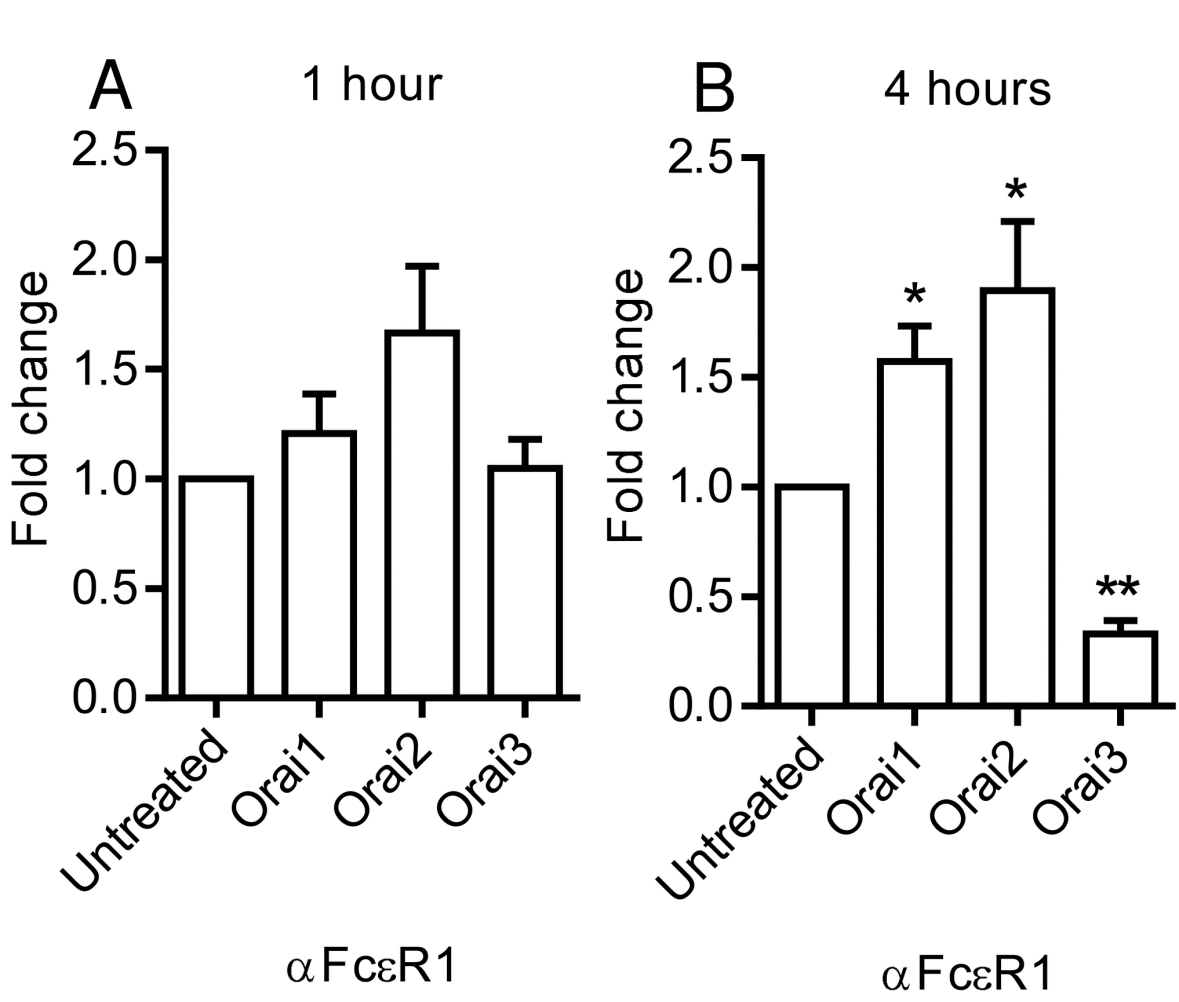
The effect of FcεRI-dependent HLMC activation on levels of Orai transcripts. Cells were activated with αFcεR1 antibody and the levels of Orai1-3 transcripts determined by quantitative RT-PCR (**A**) after activation for 1 hour and (**B**) for 4 hours. Mean ± SEM fold change compared to untreated cells. n=5, * p<0.05, ns not significant. Activation of HLMC for 4 hours results in the down-regulation of Orai3 and up-regulation of Orai1 and -2 transcripts.

Given the apparent roles of Orai1 but not Orai2 in mediator release from HLMCs, we next examined whether levels of Orai transcripts were altered following HLMC activation. Quantitative RT-PCR was performed on RNA purified from HLMCs following activation with FcεRIα antibody for 1 hour and 4 hours or from control untreated cells. Levels of Orai1 and -3 transcripts were little altered 1 hour post activation, while levels of Orai2 transcripts were slightly up-regulated though by an amount that did not reach statistical significance (Figure **6**). However, changes in the levels of Orai transcripts were more marked 4 hours following activation. Orai3 transcripts were significantly down-regulated by -3.50 ± 0.60 fold, p=0.0028, n=5 (Figure **6**). In contrast there was a small but significant up- regulation of Orai1 (1.57 ± 0.16 fold, p=0.0324, n=5) and Orai2 transcripts (1.90 ± 0.32 fold, p = 0.0271, n=5).

## Discussion

Ca^2+^ influx into human mast cells is essential for the release of mediators such as histamine, tryptase, leukotrienes and cytokines following their activation through the high affinity IgE-receptor (FcεRI). We have recently shown that members of the Orai ion channel family play a major role in FcεRI-dependent Ca^2+^ influx in human lung mast cells [15]. However the individual role if any, of each of the three members of the Orai channel family has not been defined. In this study we present evidence that Orai1 plays important roles in Ca^2+^ influx and mediator release from HLMCs. In addition, we find that levels of Orai transcripts are not static following activation – rather they appear to be actively regulated.

Our results are consistent with Orai1 but not Orai2 playing a major role in the influx of extracellular Ca^2+^ into and mediator release from HLMCs following their activation. shRNA knockdown of Orai1 significantly reduced the Ca^2+^ influx that occurs following depletion of intracellular Ca^2+^ stores by IP_3_. In addition there was a reduction of approximately 50% in both HLMC degranulation, as measured by the release of β-hexosaminidase, and of newly synthesized LTC_4_ release. In contrast shRNA knockdown of Orai2 resulted in only small reductions in Ca^2+^ influx and a marginal although significant reduction in LTC_4_ release but not degranulation. Orai2 may therefore play a minor role in Ca^2+^ influx in activated HLMCs, in keeping with its relatively lower levels of expression at the mRNA level [15].

It was not possible to interpret the results of our knockdown of Orai3. While HLMCs transduced with shRNA against Orai3 or luciferase appeared viable morphologically and by Trypan blue exclusion, the high multiplicity of infection required reduced FcεRI-dependent mediator release in the luciferase shRNA control. In addition cells became impossible to study using patch clamp electrophysiology. It is noteworthy that knockdown of Orai1 also reduced Orai3 mRNA expression although this did not reach statistical significance, and this was significantly less than the knockdown of Orai1 expression. It is therefore possible that Orai3 also contributes to HLMC mediator release following FcεRI-dependent activation. However, CRACM3 exhibits rapid inactivation during voltage steps [11], which is not evident in HLMC CRAC currents [15], suggesting that Orai1 is the dominant conducting channel. Furthermore, in spite of clear knockdown of Orai1 here, residual Orai3 channels were not evident from the electrophysiological characteristics of the residual raw currents (data not shown). A contribution of Orai3 cannot therefore be confidently excluded, but Orai1 appears to be the dominant channel mediating FcεRI-dependent mediator release from HLMCs. This is in keeping with the marked inhibition of mediator release observed from Orai1 knockout mouse mast cells [14].

We also investigated possible heteromultimerisation amongst Orai subunits by over-expression of dominant-negative pore mutations of Orai1, -2 and -3. Each Orai channel consists of a hexamer of Orai subunits, with each subunit contributing to the central Ca^2+^ conducting pore [21]. We reasoned that if for example Orai1 and Orai3 were to form heteromultimeric channels then expression of a Orai1 dominant-negative construct (E106Q) or a Orai3 dominant negative construct (E81Q) would have a greater effect on Ca^2+^ influx into and mediator release from HLMCs than if Orai1 and -3 form homomeric channels. However we found that expression of Orai1, -2 or -3 channels carrying dominant-negative pore mutations all greatly reduced Ca^2+^ influx and completely abolished mast cell degranulation. These results suggest that Orai channels potentially exist as heteromultimers in HLMCs. Since Orai2 appears to be expressed at lower levels and is less important functionally than Orai1, any heteromulitmers are likely to consist predominantly of Orai1, and possibly Orai3.

Ion channels are also implicated in cell survival. In HLMCs and the human mast cell line HMC-1, TRPM7 plays a critical role in maintaining cell survival [22]. Here, we found knocking down Orai1 or Orai2, or inhibiting Orai currents through the transduction of dominant-negative Orai constructs, had no effect on HLMC survival. This is again in keeping with data from the Orai1 knockout mouse [14].

In our previous study [15], pharmacological blockers of Orai channels reduced degranulation by approximately 50-70% in spite of completely blocking FcεRI-dependent Ca^2+^ currents in individual cells. Knockdown of Orai1 or Orai3 in this study similarly reduced degranulation by approximately 50%, although knockdown was not complete. However, our finding that over-expression of dominant-negative Orai pore mutants completely inhibited HLMC degranulation suggests that the role of Orai channels in HLMC degranulation may be underestimated by pharmacological tools and shRNA knockdown. Furthermore, these results suggest that Orai channels may be the sole Ca^2+^ influx pathway contributing to FcεRI-dependent HLMC degranulation, and that other channels postulated to contribute to this in mast cells such as TRP family members [23] are not operative in HLMC.

Our results suggest that under conditions of sustained activation, Orai isoforms may be differentially expressed, allowing the fine-tuning of Ca^2+^ influx into HLMCs. We observed a significant down-regulation of Orai3 transcripts in HLMCs following a four hour period of activation with anti-FcεRIα antibody. Orai1 transcripts were up-regulated over the same time period, though by a smaller amount. There was a similar up-regulation of Orai2 transcripts, further supporting at least some role for Orai2 in Ca^2+^ influx in HLMCs. Differential expression of Orai3 transcripts has also been observed in mammary epithelial tissue and cell lines and also in human T helper (T_H_) cells [24–26]. However in these cases, the development of cancer (mammary tissue/cell lines) or subjection of cells to oxidative stress (effector T_H_ cells) resulted in upregulation of Orai3 expression [24–26].

Because of the critical requirement for Ca^2+^ influx for mast cell mediator release, Orai channels are of great interest as potential therapeutic targets for the treatment of asthma and related allergic conditions. Because of the contribution of Orai1 to the HLMC secretory response, a greater understanding of the specific functions of Orai1 in mast cells may permit the fine-tuning of anti-Orai strategies, with the development of drugs which target these family members selectively. This in turn would prevent the off-target inhibition of Orai2 in other cells and body systems, therefore potentially reducing unwanted treatment side-effects.
